# Genetics and Breeding of *Lupinus mutabilis*: An Emerging Protein Crop

**DOI:** 10.3389/fpls.2019.01385

**Published:** 2019-10-30

**Authors:** Agata Gulisano, Sofia Alves, João Neves Martins, Luisa M. Trindade

**Affiliations:** ^1^Wageningen University & Research Plant Breeding, Wageningen University, Wageningen, Netherlands; ^2^DRAT, Instituto Superior de Agronomia, Universidade de Lisboa, Tapada da Ajuda, Lisbon, Portugal

**Keywords:** *Lupinus mutabilis*, lupin, breeding, genetics, protein crop, plant protein

## Abstract

Protein crops have gained increasing interest in recent years, as a transition towards plant-protein based diets appears pivotal to ensure global food security and preserve the environment. The Andean species *Lupinus mutabilis* emerges as an ideal protein crop with great potential for Europe and other regions with temperate climates. This species is characterized by oil and protein content similar to soybean and is highly valued for its adaptability to colder climates and low input agriculture on marginal land. However, its introduction outside the Andes has yet to take off. To date, *L. mutabilis* remains an under-studied crop, lacking high yield, early maturity and a consistent breeding history. This review paper identifies *L. mutabilis* limitations and potential uses, and suggests the main breeding targets for further improvement of this crop. It also highlights the potential of new molecular tools and available germplasm resources that can now be used to establish *L. mutabilis* as a viable protein crop.

## Introduction

Over the past decades, challenges such as food security and environmental sustainability have earned the status of main priorities worldwide and are the basis of the 17 Sustainable Development Goals (SDGs) defined by the United Nations in 2015. As world population continues to rise, our food production has already exceeded the planet’s environmental boundaries driving climate changes, biodiversity loss and unsustainable use of land and water. The growing demand for animal proteins has played an important role in this process, by turning livestock sector in the main user of agricultural land and in one of the biggest contributors to climate change. In light of the current situation, a transition from meat-intensive diets towards plant proteins-based diets is vital to ensure global food security and preserve the environment.

To create alternatives to animal protein, the cultivation of protein crops has gained interest in recent years. The European Union has launched initiatives to reduce its dependency on the import of soybean by growing an increasing quantity and variety of protein crops within the European member states. Research has focused on identifying sources of proteins that can reduce the current protein deficit while contributing to the transition to more sustainable agricultural systems. One such source is protein-rich leguminous plants. Legumes also stand out for their great potential in the reclamation of poor and marginal lands for agriculture, due to their ability to fix nitrogen and their beneficial effects on the soil (De Ron et al., 2017). Among legumes, lupins have been identified as particularly promising, characterized by high-quality protein content, suitability for sustainable production and potential health benefits (Lucas et al., 2015). The genus Lupinus includes almost 300 species, but only four play an important role in agriculture: *L. albus*, *L. angustifolius*, *L. luteus* and *L. mutabilis* (Gresta et al., 2017). The first three listed species are native to Europe and represent the majority of lupins cultivated worldwide. At the same time, despite years of extensive research, the success of these species has been hampered by unstable yields, low oil content and adaptation to a narrow-range of agro-climatic conditions. The fourth species listed, *L. mutabilis*, is a species native to the Andes, and is cultivated only in some parts of South America and not yet commercially available in Europe (Lucas et al., 2015). However, *L. mutabilis* is characterized by the highest grain quality of all cultivated lupins, with an oil content similar to soybean, and is adapted to low input farming in temperate climates. The combination of these characteristics makes *L. mutabilis* a potentially superior alternative to the current plant-based sources of protein and oil in Europe and other regions with temperate climates.

*Lupinus mutabilis* Sweet is considered to be one of the lost crops of the Incas. Its seeds are characterized by a high protein and oil content (44% dw and 18% dw, respectively), which exceeds that of any other lupin species (Blanco-Galdos, 1982; Pate et al., 1985). In addition, lupin seeds are practically devoid of starch, and the major carbohydrates found are oligosaccharides (mainly stachyose and raffinose) and cell wall storage polysaccharides (Trugo et al., 2003). Most essential amino acids, lysine in particular, are also present in the seeds (**Table 1**) together with a substantial amount of dietary fiber and fatty acids (**Table 2**) (Carvajal-Larenas et al., 2016). The history of this species as a subsistence crop in the Andes demonstrates its potential as a crop for low input agriculture on marginal lands. *L. mutabilis* shows a high adaptability to temperate and cold climates, low-fertile soils, high altitudes and harsh conditions while actively enriching the soil with nitrogen (Cowling et al., 1998). Currently, its cultivation is mostly confined to the Andean region of South America, where its bitter seeds represent a regionally important food known as *tarwi*. It is an economically accessible source of good quality protein, on par with animal proteins, to a large percentage of the population (Carvajal-Larenas, 2013).

**Table 1:** A comparison of the essential amino acids profiles (+ cystine) of four species of lupins and soybean (*Glycine Max*).

	*L. mutabilis*	*L. angustifolius*	*L. albus*	*L. luteus*	*Glycine Max*
*Histidine*	*3.5*	*2.6*	*2.0*	*3.1*	*3.8*
*Isoleucine*	*4.2*	*4*	*4.1*	*3.6*	*n.a.*
*Leucine*	*7.0*	*6.9*	*6.8*	*7.8*	*7.2*
*Lysine*	*5.8*	*4.6*	*4.5*	*4.5*	*5.4*
*Methionine*	*0.8*	*0.7*	*0.7*	*0.6*	*1.2*
*Phenylalanine*	*3.5*	*3.7*	*3.4*	*3.7*	*4.9*
*Threonine*	*3.5*	*3.4*	*3.4*	*3*	*5.4*
*Tryptophan*	*0.8*	*0.9*	*0.9*	*0.9*	*n.a.*
*Valine*	*3.8*	*3.7*	*3.8*	*3.4*	*4.9*
*Cystine*	*1.6*	*1.6*	*1.5*	*2.4*	*1.5*

Data are expressed as g/100 g of proteins (Carvajal-Larenas et al., 2016; Prakash and Misra, 1988).

(n.a., data not available).

**Table 2:** Nutritional composition of four species of Lupinus as compared to Soybean (*Glycine max*).

	*Crude protein*	*Crude lipids*	*Crude fiber*	*FA saturated/unsaturated*	Unsaturated fatty acids (g/100 g DW)			
					C18:1 (Oleic)	C18:2 (Linoleic)	C18:3 (Linolenic)	C22:1 (Erucic)
*L. mutabilis*	43.3	18.9	8.2	0.17	46.4	33.1	2.5	–
*L. albus*	38.2	11.2	8.9	0.5	54.0	18.7	8.6	0.4–2.7
*L. luteus*	42.2	5.5	15.8	0.13	28.5	48.2	6.3	*tr*-1.5
*L. angustifolius*	33.9	6.3	16	0.23	33.9	40.3	5.6	0.1–0.5
*Glycine max*	42.9	19.8	5.1	0.18	22.8	50.8	5.9–8.3	–

Data are expressed in g/100 g DW (Collins and Howell, 1957; Hudson et al., 1983; Prakash and Misra, 1988; Sharma et al., 2014; Carvajal-Larenas et al., 2016) Crude fiber: insoluble residue, primarily composed of cellulose and lignin.

(tr, in traces, less than 0.1%)

**Figure 1:**
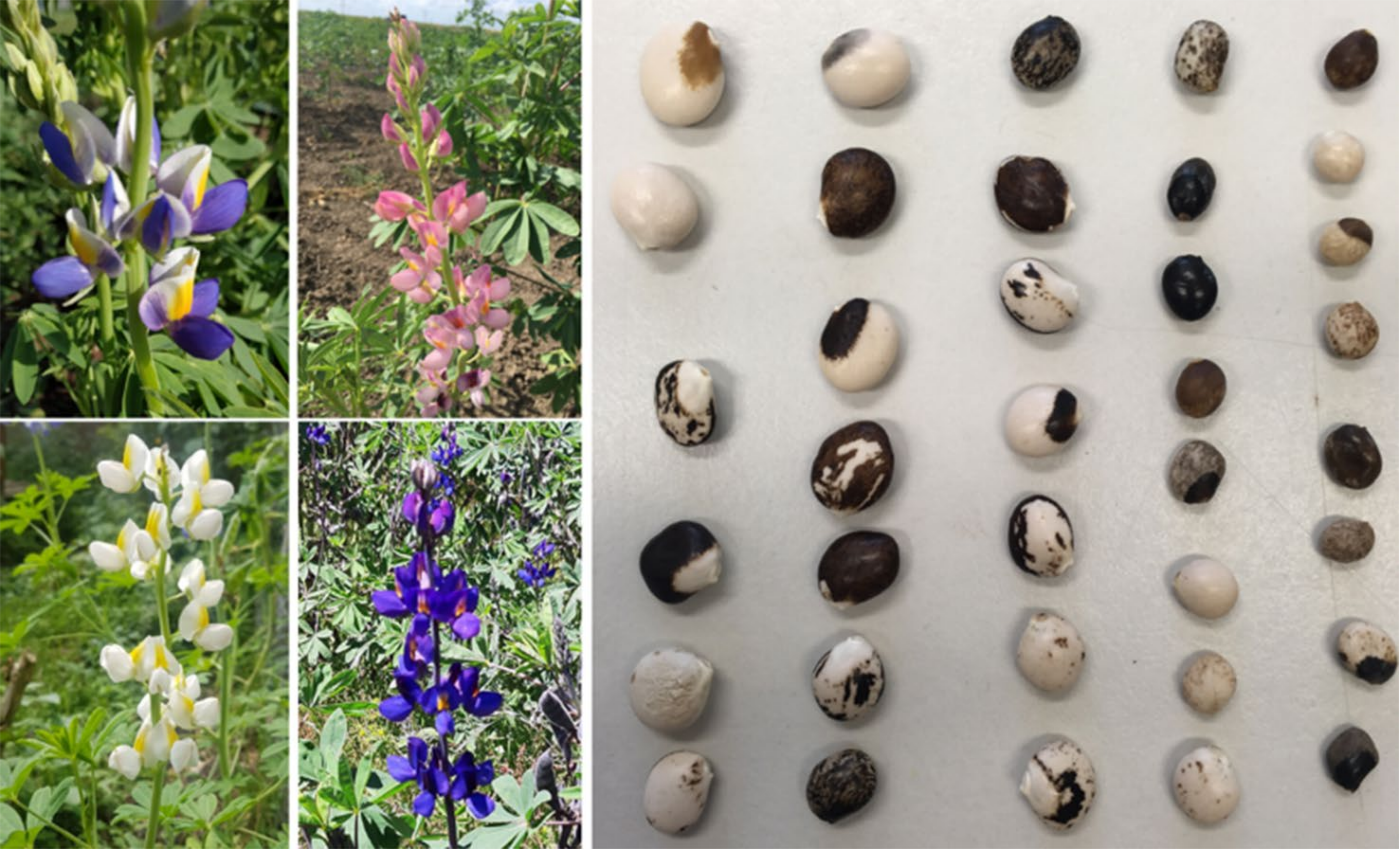
Phenotypic variation in flowers and seeds of *L. mutabilis*.

The presence of toxic alkaloids in the seeds and low yields (800–1300 kg/ha) have strongly limited the expansion of this crop (Tapia, 2015). Selection activities by Andean farmers in the past 1,500 years of cultivation have represented the only means of domestication for *L. mutabilis*, leading to semi-domesticated forms characterized by non-shattering pods, large seeds, multi-colored flowers, highly branched architecture and a more or less annual life cycle (Clements et al., 2008). It played an important role as a rotation crop in Andean agriculture, but the introduction of western pulses during the Spanish conquest in the sixteenth century, led to its decline and marginalization (Cowling et al., 1998; Caligari et al., 2000). In contrast, the wide genetic diversity that characterizes this crop has enabled its adaptation to poor soils and microhabitats, preserving its cultivation in many areas where other crops cannot grow (Carvajal-Larenas, 2013). This genetic diversity is also reflected in a broad phenotypic diversity, e.g. of seeds and flowers color (as shown in **Figure 1**).

In recent years, efforts have been made to re-establish *L. mutabilis* as a crop in South America, and to also adapt it to conditions in Europe (Caligari et al., 2000). Numerous studies investigating the nutritional profile and potential applications of these grains have found a wide range of possible products ranging from proteins, oil, and food additives to cosmetics, medicines, and bio-pesticides. In contrast, few studies have addressed the agronomic aspect of *L. mutabilis* cultivation. From these studies it emerges that the main obstacle to *L. mutabilis* cultivation is the lack of high yielding, early maturing genotypes. These results are mainly determined by an indeterminate growth habit and a lack of locally adapted genotypes, and can be overcome *via* breeding (Caligari et al., 2000). To date *L. mutabilis* remains an under-studied crop, characterized by a very young and fragmented breeding history. The important role that this crop could play in the transition toward a more sustainable food production system has prompted us to review the current state of this crop. This paper summarizes past breeding achievements and sheds light on the new breeding challenges we must resolve to establish *L. mutabilis* as a protein crop in Europe.

## Origin and Distribution of the “Andean Lupin”

The earliest archeological evidence of domesticated *L. mutabilis* seeds has been found in Mantaro Valley, central Peru and dates back to *ca*. 1800 BP. The use of RADseq in the analysis of this archeological material confirms that *L. mutabilis* was first domesticated not far from the Montaro Valley in the Cajamarca region (north Peru), from the wild progenitor *L. piurensis*. Demographic analysis suggests that *L. mutabilis* split from its progenitor around 2600 BP (650 BC) and suffered a domestication bottleneck and a subsequent rapid population expansion as it became cultivated across the Andes (Atchison et al., 2016). *L. mutabilis* presence has been reported across the eastern side of South America, from Colombia to the North of Argentina (from 10°N to 20°S), and over a wide range of altitudes, from 1,500 to 3,800 m a.s.l. (Jacobsen and Mujica, 2008). The crop is adapted to a temperate climate and is strongly influenced by day length. It is susceptible to low temperatures (−2°C) in the initial stages, and requires about 350–800 mm of rainfall and can grow for 240–300 days (Jacobsen and Mujica, 2006; Jacobsen and Mujica, 2008; Adomas et al., 2015). Based on these requirements, *L. mutabilis* could be cultivated in Southern Europe as a winter crop, and in Northern Europe as a summer crop. Nowadays *L. mutabilis* is of agricultural importance only in Ecuador, Peru, and Bolivia. Approximately 1,895 ha are cultivated in Bolivia with an average yield of 648 kg/ha, 5,974 ha in Ecuador (400 kg/ha) and 10,628 ha in Peru (1,335 kg/ha) (Mercado et al., 2018).

## Biological and Genetic Features

*L. mutabilis* is an annual herbaceous plant of the Fabaceae family. It is an autogamous species, with hermaphroditic flowers arranged in apical racemes, but characterized by a predominant level of allogamy. Different ranges of cross-fertilization by insects have been reported, fluctuating from 4–11% in Peru to 9.5–18.9% in Poland (Blanco-Galdos, 1982; Gnatowska et al., 2000). It has been observed that multiple groups of insects visit *L. mutabilis*, suggesting that this species could be a generalist; bees of the Apidae family and bumblebees from the genus Xylocopa are the main visitors in native environments, while bumblebees from the genus Bombus are more common in Europe (Ochoa-Zavala et al., 2016; Arnold et al., 2014). The isolation of different genotypes is thus indispensable in breeding programs, as much as the careful wrapping of emasculated flowers in intraspecific hybridization (Von Baer, 2011; Adomas et al., 2015). Phylogenetic analysis places *L. mutabilis* (2n = 48) within the Andean clade of Western New World species of the genus Lupinus. This genus includes almost 300 species, grouped by their different centers of origin into Old World (Mediterranean) and New World (American) subgenera. To date, *L. mutabilis* is the only cultivated species from the New World group (Gresta et al., 2017). Notably, the Andean clade to which it belongs is characterized by the highest speciation rate within the genus (Hughes and Eastwood, 2006). The species in this clade belong to a paleoploid group of plants with basic chromosome number x = 6 (Naganowska et al., 2003). Events of allo- and autopolyploidization, together with other chromosomal rearrangements, during the evolution of this species might have led to duplication/or triplication of genome regions, as observed in the Old World species *Lupinus angustifolius* (Kroc et al., 2014).

## The Unexploited Potential of *L. Mutabilis*, an Under-Studied Crop

*L. mutabilis* appears to be a valid alternative to soybeans for satisfying plant protein requirements in Europe. Like soybean, *L. mutabilis* seeds are rich in proteins as well as in oil. They can find applications as food and feed, but also as raw materials for the production of bio-based products. On the other hand, *L. mutabilis* cultivation tolerates better cold climates and can therefore contribute to the production and diversification of sustainable European sources of proteins and oil.

However, despite the clear potential, research on *L. mutabilis* has been limited. As is often the case for under-utilized crops, *L. mutabilis* has been long neglected by research and industry due to its limited economic importance on the global market. A recent domestication and a breeding history fragmented in time and space have also contributed to this neglect, resulting in a lack of genetic improvement and inferior yield. In the Andes, *L. mutabilis* germplasm collection and breeding programs started only in the 1970s and have so far relied on participatory approaches with farmers for the selection of local ecotypes (**Table 3**). The selection of genotypes with better yields mainly relies on the geographical distribution and vegetative cycle of the ecotypes and it has rarely resulted in the registration of cultivars (Fries and Tapia, 2007; Peralta et al., 2012; Vicente Rojas, 2016). In Europe, researchers began working on the selection of sweet lines in the 1920s, but it was only in the 1970s, when the nutritional value of *L. mutabilis* seeds became well known, that the interest for this crop arose. The difficult accessibility of germplasm from the Andean area was overcome in Europe with a large use of induced mutations and intraspecific crossing of mutants. Preliminary field trials of *L. mutabilis* in Europe reported large differences in seed yields, from 0.5 to 6.5 t/ha depending on years and location (Masefield, 1976; Romer and Jahn-Deesbach, 1992; Weissmann and Weissmann, 1992; Rubenschuh, 1997). In 1993, the first European project aimed at evaluating the “Adaptation of *Lupinus mutabilis* to European soil and climate conditions” was funded. Field trials reported very low seed yields (1.1 t/ha) and pointed out the need of breeding for a better plant architecture and early maturity (Caligari et al., 2000). Many years of mutation experiments in Poland have resulted in improvement in yield and sweetness and in the selection of determinate lines for research purposes, but not yet in the establishment or registration of new varieties (Galek, 2010; Galek et al., 2017) (**Table 3**). Australia has also shown interest in *L. mutabilis* and multiple projects to evaluate its potential for southern Australia were funded. Of particular relevance in their work was the selection of male sterile lines, used to introduce early vigor, anthracnose resistance, tolerance to brown spot and resistance to cucumber mosaic virus in *L. mutabilis* (Sweetingham et al., 2006) (**Table 3**). The ongoing development of recombinant inbred lines (RIL) population at the University of Western Australia is mentioned in the literature and could be exploited for mapping QTLs, however little information is available about its existence and state (J. C. Clements and M. N. Nelson, unpubl. data in Berger et al., 2013).

**Table 3:** A list of *L. mutabilis* lines involved in breeding research.

Area of Selection	Line	Characteristics	Reference
**Chile**	Inti*	Stable cultivar with 0.0075% alkaloid content in seeds, but low yield and long vegetation period.	(Gross et al., 1988; Von Baer, 2011)
**Bolivia**	Chumpi, TarwiNawi	Ecotypes grown in Potosi, characterized by dark brown seeds.	(Vicente Rojas, 2016)
	Tolarapa,Dulce	Ecotypes grown in the area of Cochabamba.	(Gross and Baer, 1981)
	Carabuco*	Variety inscribed in the National Register of seeds. Characterized by early maturing and white seeds with a cuboid flat shape.	(Vicente Rojas, 2016)
**Ecuador**	I-450 Andino*I-451 Guaranguito*	Early maturing genotypes (6 months), uniform white seeds and higher yield (1370 kg/ha on average). Susceptible to anthracnose.Registered by INIAP.	(Peralta et al., 2012)
	ECU-2700, ECU-2658	Genotypes selected for resistance to anthracnose and high yield (1445 kg/ha on average).	(Guaytarilla and Falconi, 2014)
**Poland**	KW-1	Completely determinate mutant, with no lateral branches. Characterized by tall growth, liability to lodge and low seed production.	(Römer, 1994)
	Research lines	Genotypes with shorter growth period, reduced number of branches and lower alkaloid content obtained combining intraspecific crosses with induced mutation.	(Sawicka, 1993; Stawinski and Rybinski, 2001; Galek, 2010)
**Australia**	ID13, ID18, ID32,ID33, JC243, P28725	Advanced low alkaloid, breeding lines to assess adaptation of the species to eastern states.	(Clements et al., 2008)
	P27033	Male sterile line	(Sweetingham et al., 2006)
	P25954	Restorer line	(Sweetingham et al., 2006)
	P26961	Early line	(Adhikari et al., 2012)
	P27808	Mid-season line	(Adhikari et al., 2012)
**Russia**	KVIR2381	Russian breeding line used in crosses to introduce tolerance to brown spot and resistance to cucumber mosaic virus (CMV).	(Sweetingham et al., 2006)

*L. mutabilis lines that developed into cultivars.

In Europe, the urgent need to provide alternative protein sources and recover marginal land has contributed to revive the interest in *L. mutabilis*. Recently, a new program investigating *L. mutabilis* cropping in marginal lands for enhanced bio economy has been funded under the European Union’s Horizon 2020 program (www.libbio.net). The possibility of cultivating *L. mutabilis* as a summer crop in North-central Europe and winter crop in the Mediterranean area is being investigated, along with the development of pre-industrial processing and the assessment of its socio-economic and environmental impact.

## Establishing *L. mutabilis* as a Protein Crop in EUROPE: The Breeding Challenges

### Adaptation to European Environment

The environmental differences between the native environment of *L. mutabilis* and other cultivation areas around the world, such as europe, represent one of the barriers to the expansion of this crop. In temperate climatic conditions *L. mutabilis* cultivation is characterized by a long period of maturation and uneven maturation of the pod, blossom drop, and shattering of early stage pods (Hardy et al., 1998; Swiecicki and Nawrot, 2004; Galek et al., 2007). Due to low resistance to frost during the first growth stage, sowing is limited to autumn in mediterranean environments and to spring in northern countries. In both cases, the crop will reach flowering towards the beginning of the dry season. Dry conditions can accelerate maturation, but considerably affect the biomass yield, pod set and consequently, the seed yield (Hardy et al., 1997). Hence, it is crucial to generate early maturing genotypes with increased drought tolerance and consistent yield performance (yield stability). Previous work has pointed out that vernalization has no effect on early and mid-season genotypes of *L. mutabilis*, but can reduce the flowering time of late season genotypes such as Inti. It was observed that a vernalization period of 2–4 weeks at 6°C can shorten flowering time of four weeks in Inti, reducing the gap between early- and late-flowering lines to only 3 weeks (Adhikari et al., 2012).

Research on drought stress in *L. mutabilis* has uncovered the existence of different drought tolerance strategies across genotypes, either *via* stomatal adjustments or through the accumulation of osmoprotectants. Some traits, like stomatal conductance and water potential, appear to decrease uniformly among all accessions while other traits such as membrane ion leakage or accumulation of proline and soluble sugars show particular trends depending on the genotype. This might indicate the ability of some *L. mutabilis* genotypes to adapt their cell membrane during periods of water stress, as an alternative strategy to stomatal adjustment (Lizarazo et al., 2010). Therefore, both stomatal conductance and membrane ion leakage can prove useful in the selection of drought resistant cultivars.

Response to photoperiod is another important factor for determining adaptation to different locations. Reports on photoperiodic sensitivity in *L. mutabilis* are contrasting. Hackbarth reported *L. mutabilis* as neutral to day length, while Jacobsen and Mujica affirm that in the Andean region *L. mutabilis* accelerates grain filling when the day length is short (Hackbarth, 1961; Jacobsen and Mujica, 2008).Given the latter, adaptation at higher latitudes should be based on the selection of lines less sensitive to day length effects on grain filling. A more exhaustive understanding of sensitivity to day length through characterization of germplasm collections and knowledge about its genetic basis would enable the generation of genotypes for high latitudes with little or no sensitivity (Jacobsen and Mujica, 2008).

### Growth Habit: Toward a Semi-Determinate Type

**Figure 2:**
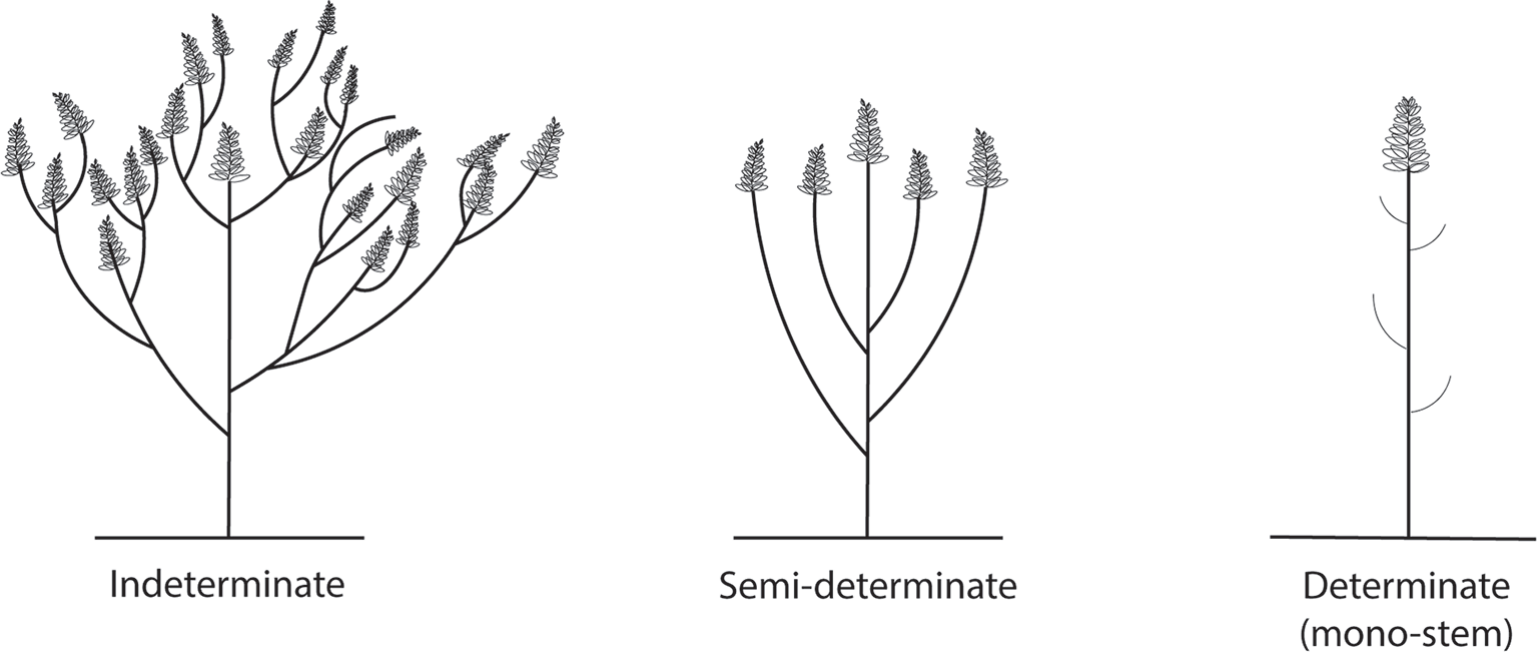
Different growth forms of *L. mutabilis*.

Indeterminate growth habit and sympodial branching pattern have been identified as the main factors limiting yield of *L. mutabilis* in European field trials (Caligari et al., 2000). In *L. mutabilis* the vegetative development begins with the production of a main stem bearing a terminal inflorescence and continues with the production of successive orders of branches throughout the entire growing season (that can be from 0 to 52 branches), as long as growing conditions are favorable (Blanco-Galdos, 1982; Hardy et al., 1997) (**Figure 2A**). This growth habit leads to an overlap of vegetative and reproductive phases, characterized in this species by a preferential partitioning of nutrients to vegetative growth. As a result, the possibility of uniform maturation is hindered and reproductive growth is constantly delayed, often so far as to coincide with late-season drought, thus further reducing productivity. Furthermore, only the racemes of the main stem and first order branches are highly productive, while the production becomes progressively weaker on the other order of branches (Adhikari et al., 2001).

It has long been proposed that the development of determinate lines could guarantee a more stable seed yield by providing an earlier and more uniform maturation (Huyghe, 1998). Determinate lupin cultivars have been obtained in *L. albus, L. luteus, and L. angustifolius* mainly through selection of spontaneous or induced mutants. Vavilov’s homologous order of restricted branching (*rb*) was selected independently in these species with a different mode of inheritance, a different number of alleles in the *rb* locus and somewhat differentiated expression in the respective species (Gorynowicz et al., 2014). Similarly, a completely determinate line of *L. mutabilis* was found upon induced mutation with EMS. This mono-stem determinate mutant—*L. mutabilis* KW 1—did not produce lateral branches, matured early and was characterized by tall stems (Sawicka, 1993) (**Figure 2C**). The inheritance of the determinate character was found to be monogenic recessive (Römer, 1994). Unfortunately, determinate plants were found to lodge and were not able to compensate for stress during main stem flowering because of increased pod set on the branches (Romer, 1995). Semi-determinate types with only one or two orders of lateral branches up to the top of the plant thus seem preferable from an agronomic point of view (Caligari et al., 2000) (**Figure 2B**). Ongoing research in Poland has focused on crossing the KW-1 mutant with early maturing mutants characterized by a reduced number of side branches for the selection of determinate form for research purposes (Sawicka-Sienkiewicz and Kadlubiec, 2001; Sawicka-Sienkiewicz et al., 2005; Galek et al., 2007). Determinate forms distinguished by medium-tall stems without lateral branches, resistant to lodgings and with early generative growth have been obtained (Galek et al., 2007). Indeterminate forms appear to have a higher mass of stems and plant aerial parts and a lower share of seeds in the yield structure, and may therefore be more suitable for biomass production (Gas, 2014; Adomas et al., 2015).

### Understanding the Mechanisms Regulating Alkaloid Content

Food and feed industries have set the strict threshold of 0.02% (DM weight) alkaloid content in lupin seeds (Cowling et al., 1998; Frick et al., 2017). Quinolizidine Alkaloids (QAs) are typically synthesized by lupin species and are mainly known for causing bitter taste and anticholinergic toxicity when present in the grains. However, QAs also play an important role in the mechanism of defense against pathogens and predators, have allelopathic functions (Wink, 1993) and constitute nitrogen reserves for the plant (Wink and Witte, 1985). They are biosynthesized from L-lysine in green tissues of the plant, transported *via* phloem and stored in all the organs of the plant, especially seeds. The content and composition of QAs depend on many factors, including genotype, biotic/abiotic stresses and pedoclimatic conditions. Each lupin species is characterized by a different alkaloid profile, known as an alkaloid-fingerprint, which fluctuates among the different organs of the plant, expressing a lower diversity and concentration in leaves than in seeds (Wink et al., 1995; Boschin and Resta, 2013). Although the chemistry of Quinolizidine alkaloids has been extensively studied leading to the identification of more than 170 structures (Wink, 1993), their biosynthetic pathway is only partially elucidated and information on the genes and enzymes involved remains limited (Frick et al., 2017).

The breeding of sweet lines of *L. mutabilis* has been mainly based on the selection of natural and induced mutants, mostly in Chile, Poland and Australia. The first stable “sweet” variety, Inti, was bred in Chile in 1980. It was characterized by an alkaloid content of 0.0075% with no reported detrimental effect on the protein (51%) or oil (16%) content, but low yield and long vegetation period hindered its adoption in different places (Gross et al., 1988; Von Baer, 2011). Yet, the inheritance of the trait was recessive and of polygenic nature, such that only 12% of the F2 plants had low alkaloid content (Von Baer and Von Baer). These characteristics require major efforts to maintain the purity of mother’s lines and to prevent the risk of progressive re-bittering due to cross-pollination in regions where lupin grows in the wild (Santana and Empis, 2001; Von Baer, 2011). In 1984, seed treatments with ethyl methanesulfonate led to the identification of the recessive allele *mutal* of the gene *Mutal*. When homozygous, the allele *mutal* was found to reduce the alkaloid level to 0.2–0.3% of seeds DM, giving rise to plants organoleptically sweet both in their seeds and vegetative parts (Williams et al., 1984). It has been suggested that along the reselection process additional minor alleles were recombined at several loci to lower alkaloid levels (Clements et al., 2008). At present, none of the mutations found has led to complete suppression of alkaloids. The reduction in total alkaloids is mainly due to a reduced percentage of sparteine and lupanine, the two most toxic QAs to humans (Williams et al., 1984). The result of the work done in Chile in the last 40 years is the acquisition of a new variety, PINTA (Inti x SCG9) which combines low content of alkaloids, high content of protein and oil, and large seeds (Von Baer, 2011). In Poland, post-mutagen treated material has been widely screened using iodine test to select 13 lines that don’t exceed 0.1% of alkaloid content in the seeds. These genotypes can be very useful in breeding programs, particularly to derive homozygous lines. In addition to facilitating the development of stables sweet varieties these homozygous lines can also be used to study the inheritance of alkaloid content in seeds (Galek et al., 2017).

A major drawback of reducing alkaloids is the increased sensitivity of plants to pests and diseases. Future work should therefore target the development of bitter/sweet lines, with sufficient level of alkaloids in the vegetative tissues to deter pathogens, but low levels in the seeds (Wink, 1990). To use this strategy fundamental knowledge on how to target the transporters involved in the translocation of QAs from source tissues to seeds is required. Candidate transporters may include plasma membrane importers in cells of reproductive tissue, and vacuolar membrane importers in cells of both aerial and reproductive tissues, as alkaloids are often sequestered within vacuoles to avoid toxic effects within tissues (Yazaki et al., 2008). To our knowledge, there are no studies yet investigating these mechanisms.

### Seed Color, a Matter of Acceptance

To further develop the market for *L. mutabilis*, it is essential to take into account consumer preferences. When whole lupin beans are marketed as food, seed coat color becomes a decisive trait for the acceptance of a cultivar. As for *L. mutabilis*, white color is the most attractive for consumers. The phenotypical diversity in seed shape and seed coat color observed in this species appears to be larger than that in all the other lupins (Blanco-Galdos, 1982). Seed characteristics with large diversity include shape (from lenticulate to spherical), primary seed color, secondary seed color and its pattern distribution (**Figure 1**). The color can vary from pearly white to solid black, and include beige/yellow, brown, dark brown and intermediate colors, like brownish green and greyish colors. Most seeds have a secondary color distribution in darker tones of the primary color. The secondary color distribution also varies between a large range of patterns, such as moustache, eyebrow, crescent, marbled, or spotted which can be expressed singularly or in combination (Falconí, 2012; Tapia, 2015). The variability in seed coat color may reflect the genetic pressure *L. mutabilis* was subjected to during its domestication, but very little is known about the genetic mechanism behind this trait. Some authors try to explain this variation hypothesizing the concerted effect of different alleles in the control of different colors and at different regions of the seed coat. That is, having different genes controlling the primary color, the secondary color, the color of the hilum and/or its adjacent region and the different patterns of distribution of secondary colors (Blanco-Galdos, 1982). Another possible explanation for the existence of such diversity in seed color and patterns may be the presence of transposable elements, as observed in other crops (Li et al., 2012). There appears to be a connection between seed color and flower color. Darker seeds lead to darker flowers, suggesting that the white color behaves as a recessive character (Blanco-Galdos, 1982).

The complexity in seed color represents a great challenge for breeders to select pure lines with uniform and heritable colors and patterns, and in particular to combine locus for high yield and white color. Still, pearly white is in 95% of the cases the most common color found in the cultivars sampled for germplasm collections of the Andean regions.

### Identification of Health-Promoting Proteins

*Lupinus mutabilis* seeds contain a high content of protein, ranging from 38 to 45% of DM; yet, the identification of unique properties in *L. mutabilis* proteins opens the door to new markets and raises the nutritional and economic value of the crop. The major protein classes encountered in legume seeds are globulins and albumins, followed by minor fractions of prolamin and glutelin (Doxastakis, 2000). Globulins (α-,β-,γ- and δ- conglutins) represent about 91–94% of the proteins in *L. mutabilis*, while albumins only ∼6.4%(Santos et al., 1997). Interest in conglutins has exponentially increased since their beneficial nutritional and pharmaceutical properties have been shown, such as cardiovascular health benefits and the use of γ- and β-conglutin in the control of insulin resistance and diabetes as well as anti-inflammatory molecules (Magni et al., 2004; Belski et al., 2011; Lima-Cabello et al., 2017). (Foley et al., 2015) used 16 individual conglutin genes previously identified in *L. angustifolius* to characterize homologous genes in five other lupin species, including *L. mutabilis*. Oddly, transcriptomic studies revealed the lowest level of conglutin transcripts for *L. mutabilis*, but the highest percentage of proteins. The expression levels for β-conglutin were particularly high (∼40%) and for γ-conglutin exceptionally low (4%), while the expression levels of α- and δ- conglutin (∼26% and 30%) were comparable to the values encountered in *L. albus* and *L. angustifolius*. Previous studies have highlighted considerable differences in structure and composition of α-conglutin and β-conglutin in *L. mutabilis* as compared to *L. albus*. In the case of α-conglutin differences were observed also within different genotypes of *L. mutabilis* (Inti and Potosi) (Santos et al., 1997), suggesting that these proteins may have different functions between and within lupin species (Carvajal-Larenas et al., 2016). In contrast, γ-conglutin was reported to possess identical composition in all lupin species studied and to represent approximately 6% of the total proteins in *L. mutabilis* seeds (Carvajal-Larenas et al., 2016). Regarding albumins in *L. mutabilis*, they were found to be less abundant and different in structure when compared to *L. albus* (Santos et al., 1997). Finally, the presence of ferritin (Fe-rich protein) in the protein profile of lupin (Strozycki et al., 2007) increases the nutritional value of this crop by offering a safe way to increase dietary iron intake. The success of its use in the development of food products for special nutritional purposes would depend on the achievement of ferritin overexpression, which may result in easier, cheaper, and more accepted methods for increasing dietary iron intake than supplementing and/or fortifying other crops (Zielińska-Dawidziak, 2015).

### Exploiting the High Nutritional Value of *L. mutabilis* Oil

*L. mutabilis* seeds are also an important source of oil. The oil content of this species (∼18%) is the highest within lupins and the only one comparable to soybean (20%). Moreover, its fatty acid composition is nutritionally superior to that of soybean: both have a similar ratio of saturated/unsaturated fatty acids (17–18%), but *L. mutabilis* has a lower amount of linolenic acid, thus avoiding the need for industrial removal of this acid as soybean and *L. albus* do, and its oil stability is naturally higher (Schoeneberger et al., 1982). In addition *L. mutabilis* oil does not have any toxic erucic acid found in other lupin species, and when compared to other edible oils presents a higher or similar quality, being inferior only to olive oil (Martins et al., 2016) (**Table 2**). Improvement of oil production *via* breeding could further enhance the economic suitability of this crop by making it dual-purpose for protein and oil, in a manner similar to the soybean (Lucas et al., 2015).

Oil content and composition are influenced by both genetic and environmental factors, and previous studies have identified a large environmental component. One study by (Williams, 1979) has reported higher oil content in late-flowering and late-maturing varieties and identified a highly significant correlation between oil content and the length of interval between flowering and pod maturity. Negative correlations between protein and oil content are also reported in the literature (r = −0.71; r = −0.77) (Perez et al., 1984; Jacobsen and Mujica, 2006; Clements et al., 2008). The identification of accessions in which oil and protein content are not (or less) inversely related could make it possible to combine high levels of both components in the seeds through selective breeding (Romer and John-Deesbach, 1986). An opportunity could come from the fiber component of lupin seeds, mainly β-galactan chains in the form of thickened cell walls of the endosperm (Al-Kaisey and Wilkie, 1992). Since catabolism of both carbohydrates and lipids generally represents the main source of germination energy, it is possible to assume that oil content might be increased *via* breeding at the expense of β-galactan content.

## Relevant Resources for Future Breeding of *L. mutabilis*

### Germplasm Collections to Exploit Natural Diversity

The Andean region, center of origin and domestication of *L. mutabilis*, represents the main hotspot of diversity for this species. Germplasm collections were started in 1974 by Dr. Oscar Blanco at the University of Cusco (Peru) and soon extended to Bolivia and Ecuador. At present, South American institutions hold more than 3,000 genotypes of Andean Lupin. The largest and most relevant germplasm collections of *L. mutabilis* are held in the gene banks of Peru, Ecuador, and Bolivia, but smaller collections are also present in Chile, Argentina, Colombia, Australia, Russia, Poland, Germany, Spain, Hungary, United Kingdom, and Portugal. Yet, reports suggest much of the diversity remains uncollected (Jacobsen and Mujica, 2008). The presence of a considerable variation across germplasm is shown by different phenotypic traits, such as a wide range of growing periods, branching patterns, color and shape of grains and flowers, and flowering times. Both Inter Simple Sequence Repeats (ISSR) and Simple Sequenced Repeat (SSR) markers have revealed a wide genetic diversity among *L. mutabilis* lines (Chirinos-Arias et al., 2015; Galek et al., 2017). In some cases, the variation illustrated by the analysis of genetic distance did not match the differences defined by morphological markers, suggesting that molecular markers other than ISSR and SSR may be more useful (Galek et al., 2017).

### Molecular and Genetic Tools Available

At present, the availability of molecular resources for breeding of *L. mutabilis* remains scarce. The majority of molecular studies have so far focused on understanding *L. mutabilis* phylogeny. Initially, isozyme numbers revealed an affinity of *L. mutabilis* to the Old World species closer than that of any other North American species studied (Wolko and Weeden, 1990). Later, the use of conserved chloroplast genes and internal transcribed spaces (ITS) highlighted the presence of an Andean group within the New World species (Käss and Wink, 1997; Ainouche and Bayer, 1999; Wink et al., 1999). Only recently, the advent of nextRADseq technology has elucidated the area and timing of *L. mutabilis* domestication (Atchison et al., 2016). Protein-based approaches have been carried out to determine seed storage protein composition in *L. mutabilis* and its differences between species and lines (Santos et al., 1997). Lately DNA based markers such as RFLP, AFLP, ISSR, and RAPD have been used to assess genetic diversity between Lupinus species and have revealed a high intraspecific variation within *L. mutabilis* populations (Olczak et al., 2001; Talhinhas et al., 2003; Zoga et al., 2008). A total of 113 SSR primers and 118 polymorphic InDel from *L. luteus* have been successfully used to characterize *L. mutabilis* genetically (Parra-González et al., 2012; Osorio et al., 2018).

Relative to other legumes, little genomic information is available for *L. mutabilis*. To date, even the number of ESTs sequenced and submitted to the genomic databases remains very low (∼65), and it mainly refers to molecular targets in ribosomal RNA (IGS and ITS) and other sequences used for taxonomic purposes [i.e. *rps16 gene*, submitted by (Keller et al., 2017)]. However, new developments in genomic technologies now provide a realistic opportunity to overcome the scarcity of genomic information and to hasten the identification of traits of interest. Over the last 15 years the limitations of approaches based on the identification of QTLs derived from biparental crosses have shifted the focus towards association mapping in large panels of diverse genotypes. Genotype-by-sequencing (GBS) techniques can now provide thousands of single nucleotide polymorphism (SNP) markers at a much lower cost than earlier techniques, and they can be used to perform genotyping studies such as Genome Wide Association Studies (GWAS). In these studies natural populations hold the potential to replace recombinant populations in gene mapping and marker-trait associations (Iqbal et al., 2012). With regard to *L. mutabilis*, GWAS could represent a possible approach to exploit the genetic resources of entire germplasm collections at once, while saving time and resources, exploiting multiple recombination events, and considering the whole allele diversity. This kind of approach may serve as a foundation study and help to identify and establish valuable genetic markers for genomic selections, which will ultimately allow informed choices for further selection of breeding material and QTL analysis.

A wider selection of tools is available for *L. angustifolius* and *L. albus*, which have been more extensively studied in the past years. Genetic maps, BAC libraries, transcriptome and proteome assemblies, QTLs and molecular markers for traits such as low alkaloids, flowering time, and anthracnose disease resistance have been developed for these species and can potentially be exploited for *L. mutabilis* improvement (reviewed in Wolko et al., 2011; Abraham et al., 2019). Furthermore, the recent release of a high-quality genome draft for *L. angustifolius (951 Mb; 2n = 40)*, and a high-quality chromosome-scale genome assembly for *L. albus* (451 Mb; 2n = 50) represent a big support for the future whole-genome analysis of other lupin species, such as *L. mutabilis* (Hane et al., 2017; Hufnagel et al., 2018). Similar 2C nuclear DNA contents were estimated in *L. mutabilis* (1.90 pg) and *L. angustifolius* (1.89 pg), suggesting that there might be a higher affinity between these two species (Naganowska et al., 2003).

## Applications and Potential Uses of *L. mutabilis*: Much More Than Proteins

*L. mutabilis* emerges as a human health food and food additive, but its potential applications go far beyond food and target the utilization of the whole plant. *L. mutabilis* seeds represent an important and versatile source of proteins. Once debittered, the seeds can be directly consumed as a snack, or as an ingredient of many products and meals. In the Andean region they are traditionally used in soups, stew and salads or as raw material for preparing flour, milk, and margarine (Falconí, 2012). Like soybean, lupins also have important applications as food ingredients in many products: lupin flour, protein concentrate, and protein isolate display physical and functional properties which are very valuable to the food and chemical sector (Carvajal-Larenas et al., 2016). These derivatives can be used as base for meat alternative or replacers, as an egg replacement, as a bread improver, as an emulsifier and to increase the nutrient content of many products. After protein extraction, the large amount of dietary fiber still available (up to 40% of seed mass in *L. angustifolius*) can find application as prebiotic and human food ingredient in the production of fiber-enriched baked goods (Clark and Johnson, 2002; Smith et al., 2006). The oil, characterized by a high nutritional value, also represents an attractive product for both nutraceutical and cosmetic purposes. Furthermore, pharmaceutics uses have also been described. *L. mutabilis* intake has been proven to reduce blood glucose and insulin levels, representing a valid alternative for treating hyperglycemic diseases (Fornasini et al., 2012). In the medical field, QAs also have an important role due to multiple properties such as anti-arrhythmic, anti-inflammatory, diuretic and hypotensive effects among others (Bunsupa et al., 2012). In addition QAs can also find application in agriculture as a bio-stimulant increasing growth and yield of other crops (Przybylak et al., 2005), as antibacterial agents (Romeo et al., 2018) or as biocidal agents replacing synthetic toxins (Bermúdez-Torres et al., 2009). Similarly a Blad-containing oligomer (BCO), a bioactive subunit of a polypeptide oligomer termed Blad (Banda de Lupinus albus doce) isolated in young cotyledons of Lupin spp. as a breakdown product of β-conglutin catabolism, has been recently introduced in the market as a novel fungicide against both human and phytopathogenic fungi, confirming the multiplicity of resources offered by this plant. BCO also acts as plant bio stimulant and exhibits bactericide activity especially towards Gram+ bacteria (Carreira et al., 2018). Beyond this, *L. mutabilis* can also be used as a fodder species. In the Andean area debittered seeds are used to feed pigs, sheep, and poultry (Cremer, 1983). However, the optimal use of the plants for feed purposes would be as a green-fodder or silage, as debittered seeds are more profitable for food applications. Uses as silage or hay for livestock feed are mentioned in the literature, but its composition and nutritional value remain unstudied (Sherasia et al., 2017). Similar to other legumes, *L. mutabilis* is also able to assimilate atmospheric nitrogen and leave appreciable amounts in the soil as post-harvest residues of up to 400 kg ha^−1^ N (Brücher, 1989; Adomas et al., 2015). Yet, it could prove more profitable to turn *L. mutabilis* biomass residues into bio-based products and energy sources, due to the boost in biomass demands in Europe.

## Future Prospects

Compared to many other pulses which dominate our agriculture (*i.e.* pea, lentil, faba bean), the domestication history of *L. mutabilis* appears very short and fragmented between Europe, South America and Australia. Even though global holdings of *L. mutabilis* represent a plethora of genetic resources, this source remains under-utilized and very often inaccessible. In addition, the lack of refined biotechnological methods in genetics, molecular cytogenetics or tissue culture, has limited the possibility of exploiting natural variability and performing distant crosses and haploidization of breeding material. The repeated use of a limited set of genetic resources in hybridization programs and the limited pre-breeding efforts account for a narrow genetic basis. Base broadening through mutation and hybridization—the main methods used so far—is a very slow process, taking many years before pure lines can be achieved. The coupled use of germplasm resources and modern approaches to broaden the genetic basis could now aid the introgression of desirable adaptive traits for specific environments, which are essential to develop *L. mutabilis* into a valuable crop outside the Andes. The selection of genotypes adapted to specific latitudes and day lengths appear fundamental for farmers both in the Andes and in other parts of the world. Indeterminate growth habit and alkaloid content still represent a main limitation, but sweet lines and determinate forms with early maturation have been generated (Galek et al., 2007) (**Table 3**). A major effort is now required to fix these traits and breed them into stable variety for agricultural purposes. Breeding targets and strategies proposed in this review are summarized in (**Table 4**). Currently, promising *L. mutabilis* lines are being screened throughout Europe aiming at the development of varieties adapted to European farming conditions within the next 10 years. Future work should focus on the development of bitter/sweet lines and on the promotion of different end-uses for proteins, oil and alkaloids which can contribute to increase the value of the crop in the near future. In this regard, studies combing genetic and multi-environment dataset will be important to unravel the genetic control of valuable traits. Further implementation of genomic selection and marker-assisted selection, will play a key role in speeding up breeding processes.

**Table 4:** Suggested breeding traits for the improvement of *L. mutabilis*, goals and proposed strategies.

BREEDING TARGETS	GOALS	PROPOSED STRATEGIES
Semi-determinate growth habit	• Determinate forms distinguished by medium-tall stems without lateral branches, resistant to lodgings and with early generative growth • Higher productivity and uniform maturation	• Identification of *rb* locus in *L. mutabilis* • Fixing the trait and breeding it into a stable variety
Environmental adaptation	• Early maturing genotypes • Increased drought tolerance • Yield stability	• Selection of early maturing genotypes Study the effect of vernalization on flowering time • Selection of genotypes based on photoperiod sensitivity • Investigation of drought tolerance strategies across genotypes • Breeding of homozygous lines
Alkaloid content	• Breeding of stable sweet varieties • Bitter/sweet lines	• Derive homozygous lines from “sweet” genotypes • Study the inheritance of alkaloid content in seeds • Study the translocation of QAs from source tissues to seeds • Target QAs transporters for the development of bitter/sweet lines
Seed color	• Seeds with uniform and heritable color (white)	• Select pure lines with uniform and heritable color patterns • Identify locus/loci responsible for color and patterns • Combine loci for high yield and white color
Proteins	• Identification and valorization of unique properties in *L. mutabilis* proteins • Increased production of γ- and β-conglutins; albumins and ferritin	• Identification of new valuable proteins • Elucidate biosynthetic pathways and functions of the different proteins
Oil	• Make *L. mutabilis* a dual-purpose crop for protein and oil	•Identification of accessions with low negative correlation between oil and protein • Elucidate relation between β-galactan and oil content in seeds

In spite of limitations, there remains enormous potential for the introduction of *L. mutabilis* as a protein crop. Its cultivation constitutes an important opportunity to provide a substantial source of protein through low input farming, both in the Andes and elsewhere in the world. In this regard, the potential of enhancing marginal lands production while contributing to the diversification of the protein market, righteously places *L. mutabilis* in the European agricultural system. Hence, *L. mutabilis* plays a major role on the protein transition scene, where plant based proteins will gradually replace animal proteins. Pivotal to achieving this aim are breeding programs focused on ensuring economic viability and consumer acceptance of the crop. Germplasm resources should be used together with conventional and molecular tools to unlock the genetic potential of *L. mutabilis* and secure it as a promising (new) protein crop. Finally, *L. mutabilis* represents a source of important traits for introduction into major lupin species or other legumes to aid their adaptation in a rapidly changing climate. Further research on this species can also provide valuable insights into important processes like protein and oil production in seeds or regulation of alkaloid content.

## Author Contributions

AG wrote the manuscript. LT coordinated the writing and revised the manuscript. SA and JM contributed to the writing. All authors approved the manuscript.

## Funding

This project has received funding from the Bio-based Industries Joint Undertaking under the European Union’s Horizon 2020 research and innovation program under grant agreement No 720726 (LIBBIO).

## Conflict of Interest

The authors declare that the research was conducted in the absence of any commercial or financial relationships that could be construed as a potential conflict of interest.
